# CRISPR/Cas12a DTR system: a topology-guided Cas12a assay for specific dual detection of RNA and DNA targets

**DOI:** 10.1093/nar/gkaf893

**Published:** 2025-09-10

**Authors:** Qingyuan Jiang, Shuqi Jin, Zhichao Qin, Junqi Zhang, Ruyi He, Zhuo Chen, Bin Qiao, Jie Qiao, Yi Liu

**Affiliations:** State Key Laboratory of Biocatalysis and Enzyme Engineering, School of Life Sciences, Hubei University, Hubei 430042, China; State Key Laboratory of Biocatalysis and Enzyme Engineering, School of Life Sciences, Hubei University, Hubei 430042, China; Pilot Base of Food Microbial Resources Utilization of Hubei Province, School of Life Science and Technology, Wuhan Polytechnic University, Hubei 430023, China; State Key Laboratory of Biocatalysis and Enzyme Engineering, School of Life Sciences, Hubei University, Hubei 430042, China; Pilot Base of Food Microbial Resources Utilization of Hubei Province, School of Life Science and Technology, Wuhan Polytechnic University, Hubei 430023, China; Department of Oral and Maxillofacial Surgery, The First Affiliated Hospital of Zhengzhou University, Zhengzhou University, Zhengzhou 450001, China; Department of Oral and Maxillofacial Surgery, The First Affiliated Hospital of Zhengzhou University, Zhengzhou University, Zhengzhou 450001, China; Pilot Base of Food Microbial Resources Utilization of Hubei Province, School of Life Science and Technology, Wuhan Polytechnic University, Hubei 430023, China; State Key Laboratory of Biocatalysis and Enzyme Engineering, School of Life Sciences, Hubei University, Hubei 430042, China; BravoVax Co., Ltd, Wuhan, Hubei 430075, China

## Abstract

The CRISPR/Cas12a technology has revolutionized molecular diagnostics. However, existing Cas12a systems depend on continuous target DNA activation, which limits them to single-target detection. In this study, we developed a novel topology-guided Cas12a system, the double-target responsive (DTR) system, capable of being activated by noncontiguous dual RNA/DNA targets. The DTR system employs two split CRISPR RNA (crRNA) fragments and two Cas12a proteins that cooperatively reconstitute upon recognizing two nucleic acid activators. We demonstrated the DTR system’s ability to specifically detect dual nucleic acid substrates in a single readout, achieving a detection limit of 78 fM for RNA and exceptional specificity for single-nucleotide variations. Additionally, we successfully applied the DTR system to clinical samples, enabling simultaneous detection of two oral squamous cell carcinoma-related microRNAs (miR-155 and miR-let-7a), thereby distinguishing healthy individuals from patients. This work establishes an efficient Cas12a-based platform for sensitive, simultaneous, and discriminative detection of RNA and DNA targets, enhancing the versatility of Cas12a in analytical detection and clinical diagnosis.

## Graphical Abstract

**Graphical Abstract:**
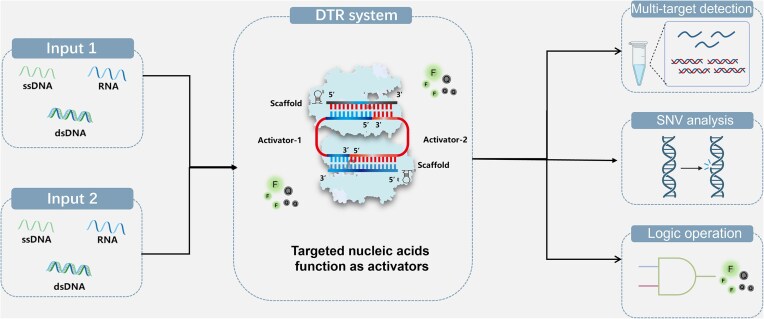

## Introduction

The emergence of CRISPR (clustered regularly interspaced short palindromic repeats)/Cas systems has profoundly transformed molecular biology, providing unprecedented precision in genome editing and regulation [1, 2]. Among the diverse Cas proteins, Cas12a, a type II-C RNA-guided DNA endonuclease, stands out for its unique capabilities [3–5]. This enzyme not only performs site-specific cleavage, known as c*is*-cleavage, on target double-stranded DNA (dsDNA), but also exhibits *trans*-cleavage activity on any single-stranded DNA (ssDNA) upon recognizing a complementary sequence within the spacer region of the crRNA. This dual functionality has facilitated the development of highly sensitive and specific nucleic acid detection platforms, such as DETECTR [3] and HOLMES [6], highlighting their potential to enhance point-of-care diagnostic tools.

Previously, Cas12a-based detection tools have primarily focused on DNA analysis, with the exception of methods that incorporate reverse transcription for RNA detection [7, 8]. Recently, we developed the SCas12a [9] and SCas12aV2 [10] assays, which integrate the Cas12a enzyme with a split CRISPR RNA (crRNA) system comprising scaffold RNA and spacer RNA and enable sensitive and direct RNA detection without the need for reverse transcription. However, all existing Cas12a detection systems depend on the activation mechanism triggered by the recognition of contiguous DNA or RNA substrates, limiting them to single-target detection. To achieve multiplexed target detection, additional complex logic gates [11, 12], physically segregated microfluidic technology [13], and dynamic molecular circuits involving other enzymes [14, 15] are typically required. Nevertheless, several logic gate designs suffer from issues such as slow reaction kinetics, high structural complexity, and significant signal leakage [16–18]. Recently, Zhao *et al.* developed a CRISPR–Cas13a Gemini system where two independent target RNAs co-activate dual Cas13a nucleases [19], generating a positive signal only when both RNA targets are simultaneously present. However, the Gemini system is limited to sensing microRNA (miRNA) substrates and is incapable of performing specific AND-logic detection for DNA and DNA/RNA mixed substrates.

**Figure 1.**
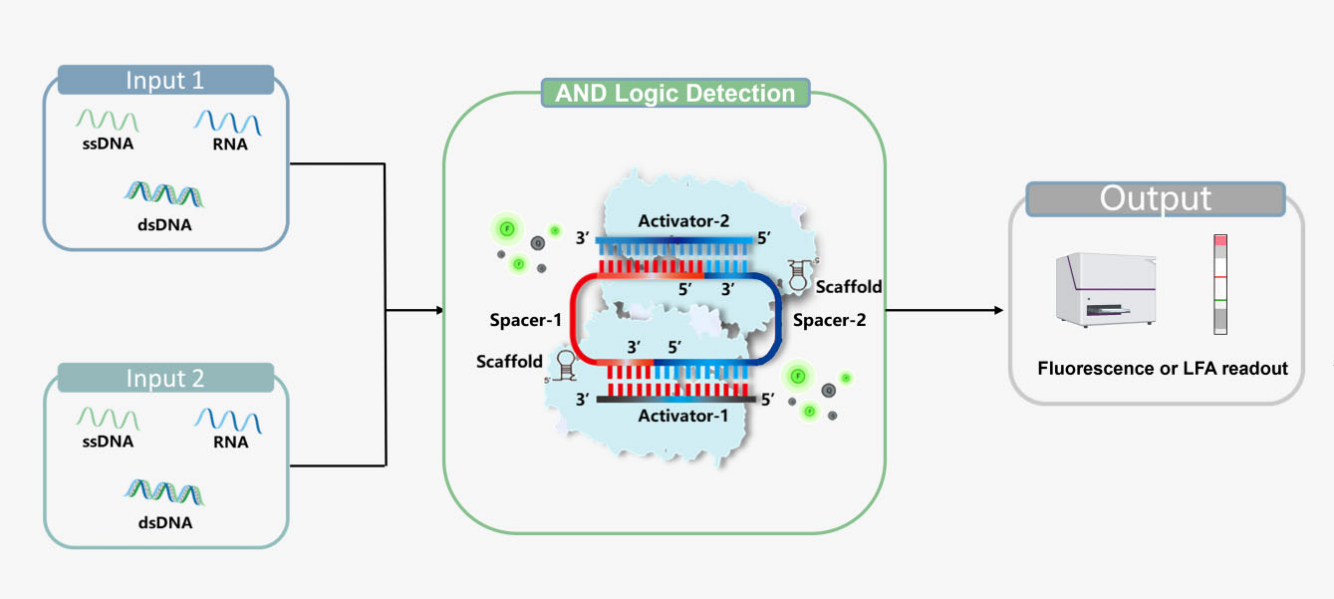
Schematic illustration of the Cas12a DTR system. The restricted AND-gate logic for dual RNA and DNA target detection.

In this work, we developed a novel topology-guided Cas12a system, termed the double-target responsive (DTR) system, for noncontiguous target RNA/DNA activation. In this system, two split crRNA fragments are cooperatively reconstituted with two Cas12a proteins into a functional conformation upon recognition of two nucleic acid activators (Fig. 1). We validated the universality of the DTR system by demonstrating its ability to perform specific dual detection of various types of nucleic acid targets in a single readout. Additionally, the DTR system exhibits exceptional specificity for single-nucleotide variations (SNVs) across all detection sites of an RNA target. Finally, we successfully demonstrated the application of the DTR system to clinical samples for the simultaneous detection of two oral squamous cell carcinoma (OSCC)-associated miRNAs (miR-155 and miR-let-7a) in a one-pot assay, effectively distinguishing between healthy individuals and patients with OSCC.

Taken together, we have developed a highly efficient Cas12a-based detection platform that enables sensitive, simultaneous, and discriminative detection of both RNA and DNA targets, thereby significantly enhancing the versatility of Cas12a in analytical detection and clinical diagnosis.

## Materials and methods

### Ethical statement

For this study, clinical human OSCC resection specimens for miRNA detection were collected by the Department of Oral and Maxillofacial Surgery at the First Affiliated Hospital of Zhengzhou University, with approval from the ethics committee.

### Materials

6-Carboxyfluorescein (FAM)-labeled ssDNA and RNA probes, as well as all the DNA and RNA oligonucleotides (Supplementary Tables S1–S2), were synthesized by Sangon Biotech (Shanghai, China). Buffer preparation reagents were purchased from Sinopharm Chemical Reagent Co., Ltd (Beijing, China). NEB Buffers 1.1, 3.1, 4, and CutSmart were supplied by New England Biolabs (Beijing, China). The miRCURY LNA RT Kit was obtained from Qiagen, while SuperScript™ IV Reverse Transcriptase was acquired from Invitrogen™. RNase H, RNAPol buffer, ribonucleotide mix, murine RNase inhibitor, and T7 RNA polymerase were also sourced from NEB. DNase I-XT for RPA amplicon treatment was obtained as required. Proteinase K (Takara, Dalian, China) was used for protein degradation. Ultrapure water was utilized in all experiments. All DNA/RNA samples were dissolved in Diethylpyrocarbonate (DEPC)-treated water and stored at −20°C.

### Expression and purification of recombinant Cas12a ortholog proteins

Synthetic genes encoding AsCas12a and LbCas12a were cloned into the pET-28a(+) vector. Plasmid integrity was confirmed by restriction enzyme digestion and sequencing prior to transformation into *Escherichia coli* BL21 (DE3). A single colony was cultured in LB medium with 100 μg/ml kanamycin overnight at 37°C. The next day, the culture was diluted to OD_600_ = 0.8 in 1 l of Terrific broth (TB), chilled on ice for 10 min, and induced with 0.5 mM Isopropyl-beta-D-thiogalactopyranoside (IPTG) at 18°C for 16 h. Cells were harvested by centrifugation, lysed in buffer A (20 mM Tris–HCl, pH 7.5, 300 mM NaCl, 1 mM PMSF, 5 mM β-mercaptoethanol) with protease inhibitors, and purified using Ni-NTA IMAC followed by size-exclusion chromatography on a HiLoad^®^ 16/600 Superdex^®^ column. Purified proteins were concentrated with a 100 kDa MWCO filter, quantified by Bradford assay, flash-frozen in liquid nitrogen, and stored at −80°C.

### Gel electrophoresis

**Figure 2.**
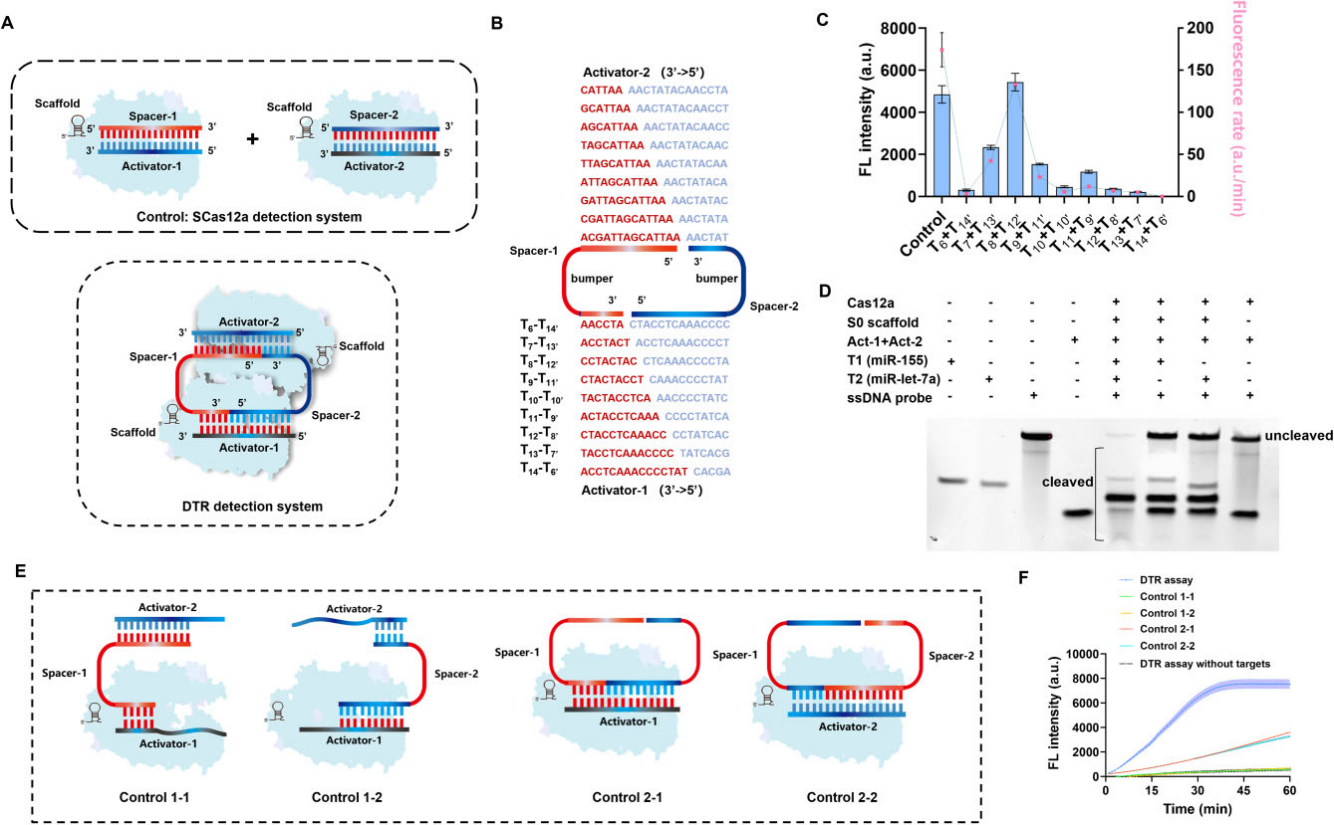
Cas12a DTR System for noncontiguous target RNA activation. (**A**) Schematic of SCas12a and DTR detection systems for activating Cas12a. (**B**) Schematic representation of nine break sites (sites 6–14) located in the spacer region, along with the corresponding nine groups of ssDNA activators. The red and blue colors indicate the activator regions that are complementary to the two target miRNAs (Spacer-1 and Spacer-2), respectively. (**C**) Comparation of target miRNAs-mediated Cas12a *trans*-cleavage efficiency at each combination. The control represents the data of SCas12a assay targeting the same combination of miRNAs. Data are presented as mean values ± standard deviation from three independent experiments. (**D**) Electrophoretic gel analysis of Cas12a activation by T8–T12′. The experiment was independently repeated twice with consistent results. Source data are provided in the Supplementary data. (**E**) The formation of single Cas12a RNP either with two ssDNA activators and one spacer RNA (Control 1–1, Control 1–2) or in the presence of two spacer RNAs and one ssDNA activator (Control 2–1, Control 2–2). (**F**) Fluorescence analysis of the DTR assay and Control 1–1, 1–2, 2–1, and 2–2. The Cas12a DTR system had the highest fluorescence intensity.

Initially, the Cas12a DTR systems were constructed in reaction buffer by mixing one of the Cas12a nucleases with its corresponding split crRNA at an equimolar ratio. This was followed by a 20-min incubation at room temperature. Subsequently, the *trans*-cleavage activity of the reconstituted Cas12a ribonucleoprotein (RNP) complex was assessed under the following conditions: a 10 μl reaction mixture comprising 1 μl of 10× NEB buffer 2.1, 40 nM Cas12a, 40 nM split RNA (20 nM miRNA-1 and 20 nM miRNA-2), and 20 nM of a 42-nucleotide ssDNA reporter in the presence of 40 nM ssDNA activators (20 nM ssDNA-1 and 20 nM ssDNA-2). The reactions were incubated at 37°C for 60 min and then terminated by heating at 90°C for 10 min. The resulting products were resolved on a 12% polyacrylamide gel (Fig. 2D), using 1× Tris-Borate-EDTA (TBE) as the electrophoresis buffer at a constant voltage of 100 V for 120 min. Finally, the gel was stained with GoldView and visualized under a UV transilluminator.

### Application of the DTR fluorescence and LFA assay for RNA detection

The DTR assay was carried out on the CFX96 Touch Real-Time PCR System (Bio-Rad, CA, USA) for fluorescence detection. Cas12a–crRNA complexes were prepared by mixing 500 nM Cas12a RNP with 1× NEB buffer 4, nuclease-free water, and 1 μM Polyvinyl pyrrolidone (PVP), followed by a 10-min incubation at room temperature. Auxiliary DNA activators (ssDNA, pseudo-hybrid DNA, and dsDNA) were subsequently added to the complexes. Each reaction consisted of 1 μM Fluorescence Quencher (FQ) reporter, target RNA at the appropriate concentration, and had a total volume of 20 μl. Reactions were incubated at 37°C for 20 min, and changes in fluorescence at 520 nm were monitored. Additionally, the same assay was performed using Lateral flow assay (LFA) detection (GenDx Cas12a detection kit, Suzhou, China).

### Clinical sample detection by DTR assay and RT-qPCR

Total RNA was extracted from tumor resection specimens of seven oral cancer patients and three healthy controls using the RNAprep Pure Micro Kit (TIANGEN, Beijing, China). The RNA was then used in a 20 μl DTR assay reaction to detect miR-155 and miR-let-7a. For Reverse transcription polymerase chain reaction (RT-qPCR) analysis, stem-loop primers and the Hairpin-it miRNA Detection Kit (GenePharma, Suzhou, China) were employed. RNA quantification followed the manufacturer’s protocol, and a standard curve was generated to correlate miRNA concentrations with Cq values. Levels of miR-155 and miR-let-7a in clinical samples were determined using this curve. RT-qPCR was performed on a CFX96 Touch Real-Time PCR System (Bio-Rad, USA) with SYBR Green detection. Thermal cycling included initial denaturation at 94°C for 3 min, followed by 40 cycles of 94°C for 12 s and 62°C for 30 s.

### Limit of detection calculation

The detection limit (LoD) was established by performing the *trans*-cleavage assay with different concentrations of RNA or DNA targets. The LoD value was computed using the formula 3*σ*/slope, in which *σ* represents the standard deviation of three blank measurements.

### Statistics and reproducibility

No statistical method was used to predetermine the sample size. All data were incorporated into the analyses without exclusion. The experiments were not randomized. Moreover, the investigators were informed of the allocation during the experiments and outcome assessment.

## Results

### Noncontiguous target RNA activation through the Cas12a DTR system

Recently, we have developed a split crRNA-based Cas12a detection method termed SCas12a for the direct detection of RNA targets [9]. However, this assay is not effective for simultaneous detection of two RNA targets in one pot (Fig. 2A), as either of them can initiate the *trans*-cleavage activity of Cas12a. To overcome this issue, we first designed a topology-guided Cas12a system, termed DTR, for noncontiguous target RNA activation (Fig. 2A). According to the design, the two target miRNAs are intended to function as spacer RNAs. Each of them comprises two ssDNA activator binding sites and a 2-nt bumper domain. This structural arrangement is expected to enable the simultaneous activation of both Cas12a nucleases.

For proof-of-concept study, we selected miR-let-7a and miR-155 as Spacer-1 and Spacer-2, which consists of 22 and 24 nucleotides, respectively. To investigate whether the break sites of the two spacers influence the catalytic activity of Cas12a, we designed nine combinations in which the break sites ranged from 6 to 14 (Fig. 2B). Additionally, the SCas12a detection system targeting these two miRNAs was employed as a control group (Fig. 2A). The fluorescence kinetic assays revealed that the combination (T8 + T12′) achieves the highest activation, exhibiting performance comparable to that of the SCas12a assay (Fig. 2C). The electrophoretic gel analysis further confirmed the effectiveness of the T8 + T12′ group (Fig. 2D). In addition, our results indicate that the presence of a single spacer (Fig. 2E, Control 1–1 and Control 1–2) is insufficient to activate the DTR system (Fig. 2F). Furthermore, the inclusion of a single activator (Fig. 2E, Control 2–1 and Control 2–2) results in only partial activation of the DTR system (Fig. 2F). These results provide evidence to support the notion that the Cas12a DTR system functions as a dimer, as proposed in Fig. 1A. Interestingly, the T12 + T8′ group, which exhibits the same break length but involves cleavage sites on distinct RNA substrates, demonstrates only marginal increases in fluorescence relative to the DTR system (Fig. 2C). This finding further suggests that the correct assembly of functional Cas12a dimer RNPs is critical for the DTR system.

To exclude potential sequence bias, we next examined various miRNA combinations (Supplementary Fig. S1). Consistent results were obtained, indicating the universality of this assay for dual miRNA detection. Moreover, we dissolved the completely assembled Cas12a DTR system and checked it by 120 KeV Cryo-electron microscopy (cryo-EM). Many particles contain two Cas12a nucleases positioned next to each other (Supplementary Fig. S2A), with a first-to-first architecture, indicating the formation of complex as anticipated. In contrast, the cryo-EM images of Control 2–1 or Control 2–2, which contain one set of Cas12a RNPs, show much smaller sizes and exhibit single-protein morphology (Supplementary Fig. S2B). Finally, we conducted Dynamic Light Scattering (DLS) experiments, and the data (Supplementary Fig. S3) reveal that a significantly larger complex is formed in the DTR system compared to the Cas12a protein alone and the complex in Control 2–1.

Collectively, these results demonstrate that the precise topological reconstitution of two active Cas12a complexes is essential for the functionality of the DTR detection system.

### Synergistic activation mode for differential concentration of miRNA substrates

**Figure 3.**
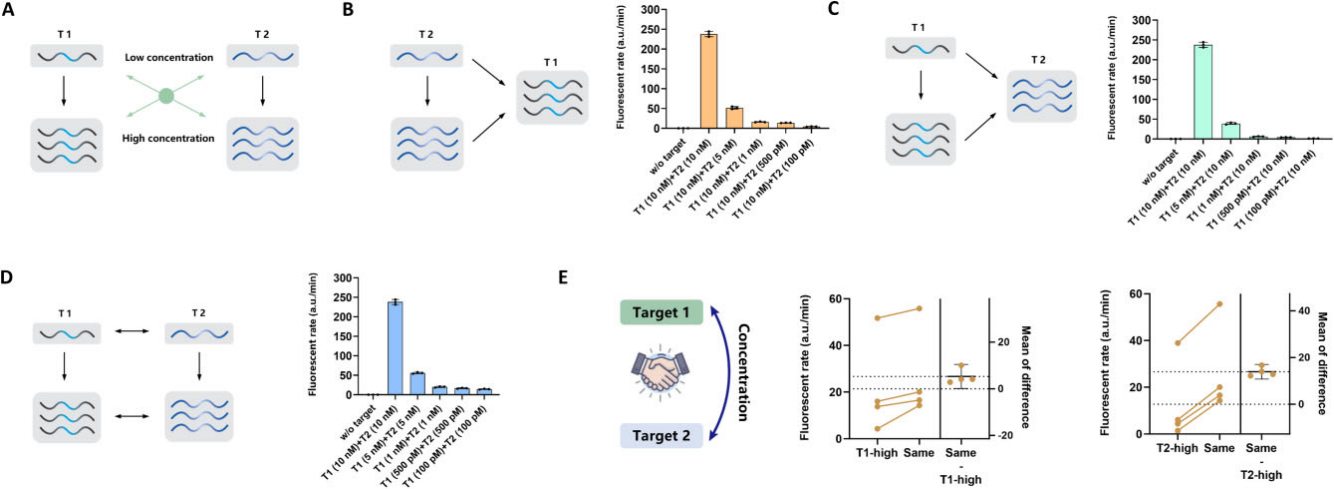
Dual-input dependency of the Cas12a DTR system. (**A**) Synergistic activation effects for differential concentration of T1 and T2. (**B**) A fixed T1 concentration with different T2 concentrations. (**C**) A fixed T1 concentration with different a2 concentrations. (**D**) The same concentration for T1 and T2. (**E**) Comparison of fluorescent rate for different concentration activation modes.

To determine whether Cas12a activity in the DTR system depends on the cooperative recognition of both targets, we established concentration gradients for miRNA substrates (T1 and T2) and evaluated their synergistic activation effects (Fig. 3A). Specifically, we anchored 10 nM of T1 and paired it with progressively decreasing concentrations of T2 (10 nM, 5 nM, 1 nM, 500 pM, and 100 pM). As the concentration of T2 decreased, the fluorescence rate markedly diminished, becoming negligible at T2 concentrations below 500 pM (Fig. 3B). A comparable trend was observed when the roles of T1 and T2 were interchanged, confirming that both components are indispensable for efficient activation (Fig. 3C). Furthermore, as a control, we tested equal concentrations of T1 and T2 ranging from 10 nM to 100 pM, which elicited a significantly stronger response within the same concentration groups (Fig. 3D). The results demonstrate that in the context of synergistic activation, the enzymatic activity of Cas12a is modulated by the combined concentrations of both activators rather than being strictly dictated by the lowest concentration of a single activator, indicating a concentration threshold and cooperative regulatory behavior (Fig. 3E). These findings emphasize the critical role of topological assembly and dual-input dependency in the DTR system, laying a foundation for downstream logic operations and experimental designs.

### Specific dual detection of RNA and DNA targets using the DTR assay

**Figure 4.**
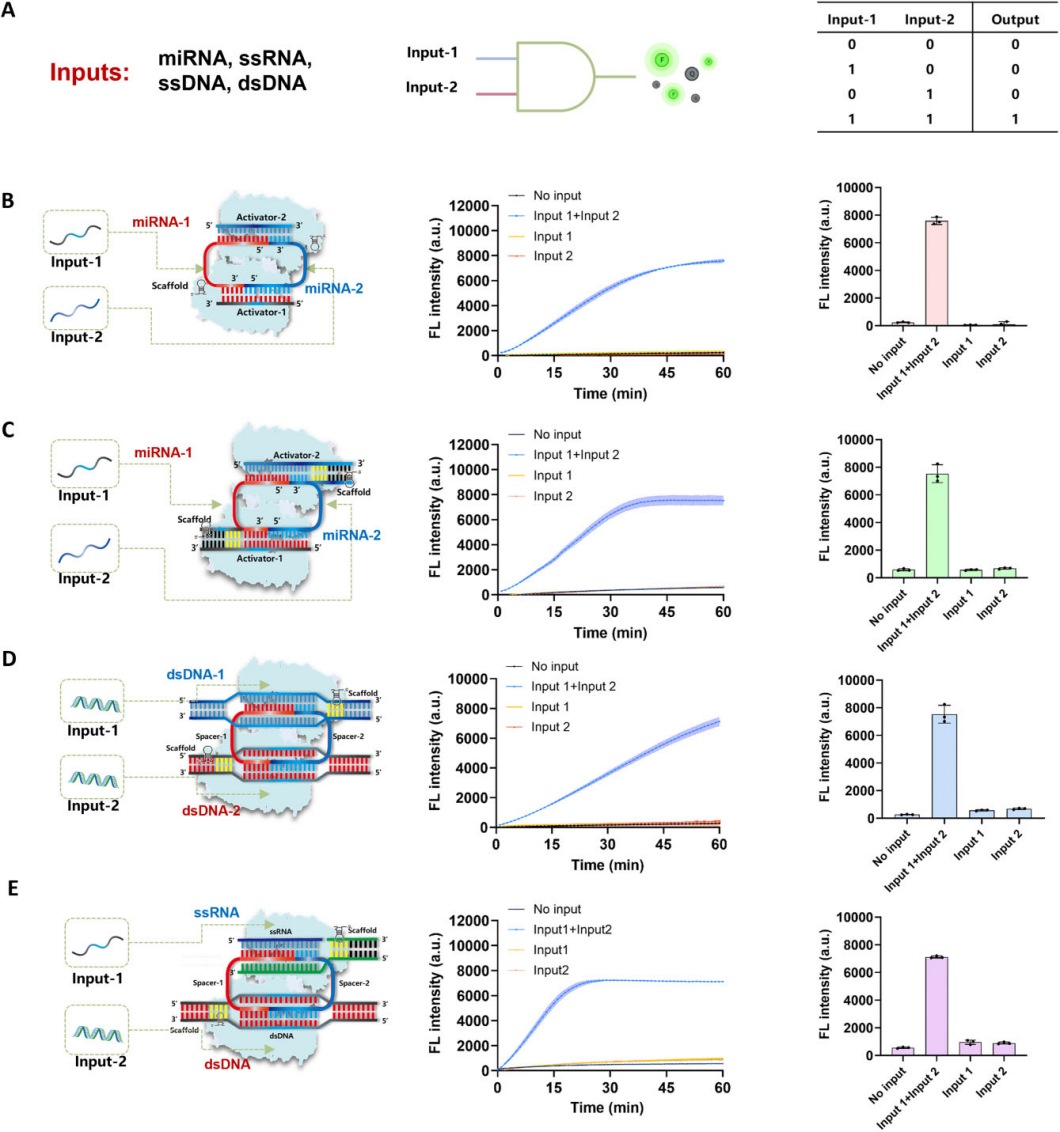
Specific dual detection of nucleic acids by Cas12a DTR system. (**A**) Schematic representation of the DTR assay for AND-logic sensing of two nucleic acid inputs. (**B**) Fluorescence analysis for the simultaneous detection of two miRNA inputs by DTR assay using two ssDNA activators. (**C**) Fluorescence analysis for the simultaneous detection of two miRNA inputs by DTR assay using two hybrid dsDNA activators. (**D**) Fluorescence analysis for the simultaneous detection of two dsDNA inputs by DTR assay. (**E**) Fluorescence analysis for the simultaneous detection of one ssRNA and one dsDNA input by DTR assay. Data are presented as mean values ± standard deviation from three independent experiments.

Boolean logic can process various types of information represented by 0 (NO) and 1 (YES), making it highly valuable for qualitative analysis [20, 21]. Here, we anticipated that the designed Cas12a DTR system could directly integrate input signal recognition, signal decoding, and effector output, enabling precise AND-logic detection of dual nucleic acid inputs (Fig. 4A). Firstly, we optimized logic gates specifically for miRNA and ssDNA detection. As shown in Fig. 4B, the two target miRNAs were designated as Input-1 and Input-2. The results demonstrated that Cas12a could only be effectively activated when all inputs precisely matched the supplied ssDNA activators, thereby forming a fully assembled closed-loop structure. In cases of absent or incomplete input signals, Cas12a activation was significantly suppressed due to incomplete structural assembly. Moreover, this strategy can be also applied for specific dual detection of ssDNA molecules when they serve as target nucleic acid inputs by providing the corresponding spacer RNAs. The DTR system is primarily designed for specific AND-logic detection; however, it can also be adapted for OR-logic detection of nucleic acids. For instance, in the detection of two miRNAs (Supplementary Fig. S4), two complementary activator ssDNAs were used instead of the spacer RNAs shown in Fig. 4B. Under these conditions, the presence of either Input-1 (miRNA-1) or Input-2 (miRNA-2) is sufficient to trigger Cas12a’s *trans*-cleavage activity.

Recently, we developed the "Pseudo hybrid DNA-RNA" (PHD) assay by using a protospacer adjacent motif (PAM)-containing hybrid DNA activator for direct RNA sensing by Cas12a [22]. In this work, we utilized the same hybrid DNA activator (Fig. 4C) for the specific dual detection of two miRNA inputs, achieving a higher kinetic rate compared to the ssDNA activator. This design offers an additional efficient strategy for subsequent experimental planning. In the next step, we examined the applicability of the DTR system for dual dsDNA target detection. By using two independent dsDNA substrates as inputs (Fig. 4D), we evaluated their impact on Cas12a activation efficiency. The results demonstrated that, despite a slight reduction in kinetic rate caused by the inherent structural redundancy of dsDNA, the DTR system still reliably detected dual dsDNA targets. Finally, we successfully extended the DTR system to the AND-gate logic detection of RNA and DNA substrates (Fig. 4E). Through the rational design of activator and target nucleic acid sequences, we achieved, for the first time, the specific simultaneous detection of ssRNA and dsDNA.

Collectively, these findings highlight the advantages of the DTR system in integrating diverse nucleic acid signals and enabling topologically driven multitarget recognition and cooperative activation, thereby emphasizing its broad applicability and potential in nucleic acid multiplex detection.

### Determination of the sensitivity and specificity of the DTR assay

**Figure 5.**
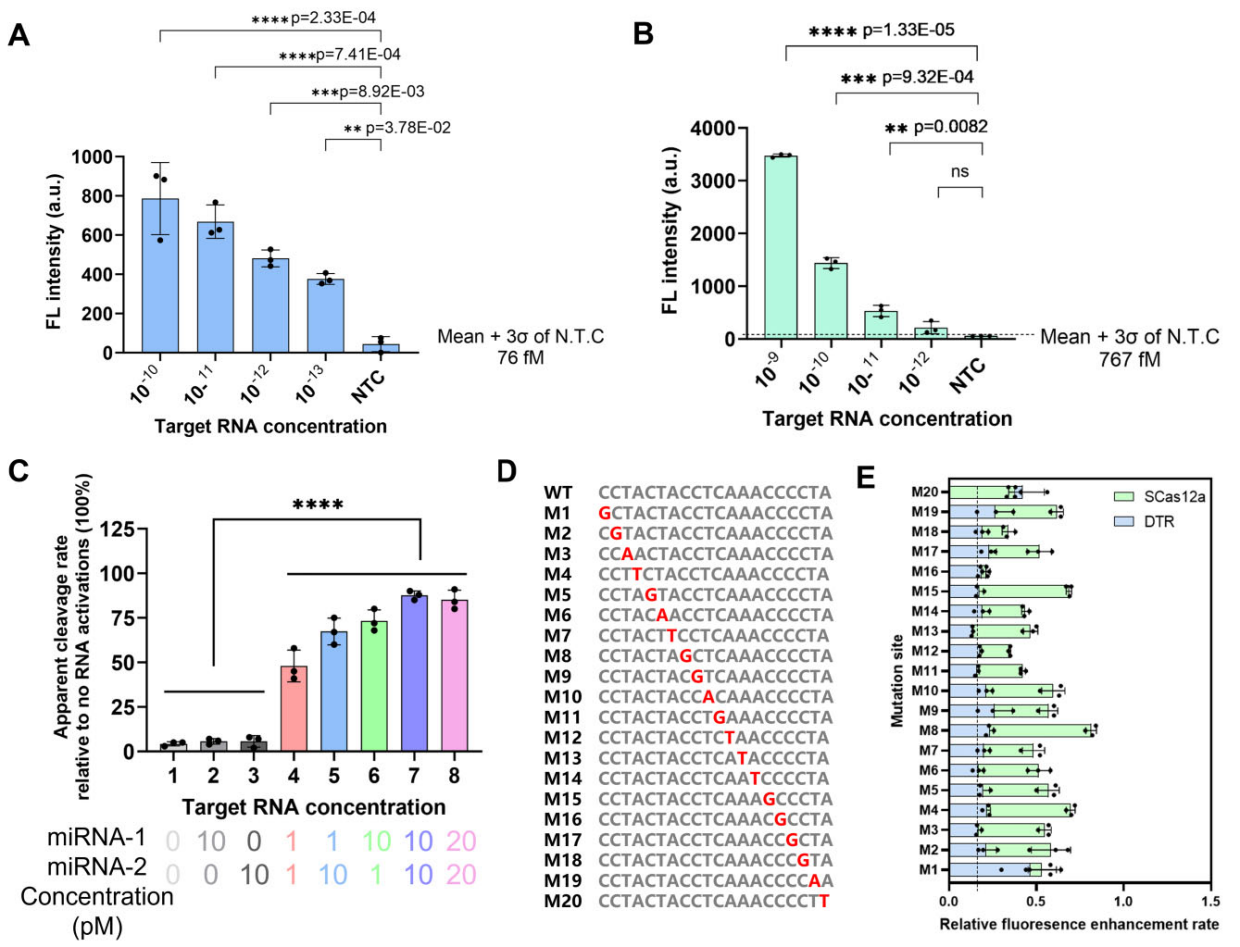
Determination of sensitivity and specificity of the DTR assay. (**A**) The limit of detection for miRNA targets using the DTR system with ssDNA activators was determined by a fluorescence assay. (**B**) The limit of detection for miRNA targets using the DTR system with hybrid DNA activators was determined by a fluorescence assay. The plot illustrates the background-subtracted fluorescence intensity at *t*= 60 min for varying concentrations of the target. All the experiments were conducted in triplicate and error bars represent mean value +/− SD (*n* = 3), and statistical analysis was conducted using a two-tailed *t*-test. Statistical significance was determined as follows: ns (not significant) for *P* > .05, * for *P* ≤ .05, ** for *P* ≤ .01, *** for *P* ≤ .001, and **** for *P* ≤ .0001. (**C**) The Cas12a DTR system enables the distinction of samples containing two RNA targets with varied concentrations and ratios from both negative controls and samples containing only one RNA target, as analyzed by a two-tailed *t*-test. Data are presented as mean values ± standard deviation from three independent experiments. (**D**) ssDNA activators were designed with point mutations across the length of the pairing region in an miR-155 target. The mutation location is identified by “M” following the nucleotide number where the base has been changed to its complementary nucleotide (3′–5′ direction). (**E**) Comparison of fluorescence fold changes in the *trans*-cleavage assay between SCas12a and DTR utilizing ssDNA activators. All fluorescence values were normalized to those of WT activator. Statistical analysis for *n* = 3 biologically independent replicates comparing the normalized fold change for SCas12aV versus DTR assay. Statistical analysis was conducted using a two-tailed *t*-test. Statistical significance was determined as follows: ns (not significant) for *P* > .05, * for *P* ≤ .05, ** for *P* ≤ .01, *** for *P* ≤ .001, and **** for *P* ≤ .0001. Error bars represent mean value +/− SD (*n* = 3).

We first evaluated the detection sensitivity of the DTR system for miRNA targets using the two strategies shown in Fig. 4B and C. By maintaining the ratio of the two miRNAs at 1:1 and varying their total concentration, the LoD of the DTR system was measured to be 78 fM (Fig. 5A) for the ssDNA activator and 767 fM for the hybrid DNA activators (Fig. 5B). The femtomolar-level sensitivity of the DTR system is comparable to that of previously reported Cas12a-based systems for miRNA detection [23–26]. Because ssDNA is simpler and more cost-effective, we adopted the first design strategy (Fig. 4B) for subsequent miRNA detection studies.

Next, we aimed to test more biologically relevant situations; i.e. the two target miRNAs usually exist in different ratios. We found that the DTR assay could significantly distinguish samples containing two miRNA targets with varied concentrations (1, 10, and 20 pM) and ratios (1:10 and 10:1) from negative controls and samples containing only one RNA target. However, no significant difference was observed when the concentrations of the two RNA targets were both ≥1 pM, regardless of their ratio (Fig. 5C). Additional experiments conducted at varying ratios further supported this conclusion (Supplementary Fig. S5), although the fluorescence intensity was lower at higher concentrations. These results confirmed the previously demonstrated synergistic activation of the DTR system and laid the foundation for clinical sample testing.

Beyond sensitivity, target recognition specificity is an even more critical criterion for evaluating nucleic acid detection methods. Given the stringent requirements for the assembly of components in the DTR system, we hypothesized that the DTR assay might exhibit superior specificity toward SNVs. To evaluate the specificity of the DTR assay for RNA substrates, we maintained miRNA-2 with constant sequences and designed a series of 20-nt target sequences in miRNA-1, each incorporating a single mismatch relative to the fully complementary sequence (Fig. 5D). The specificity of target recognition was evaluated by calculating the fluorescence ratio between mismatched and fully matched targets. A lower ratio indicates better discrimination of mismatched targets. Unexpectedly, the results demonstrated that the DTR system maintains high specificity for SNVs at all tested detection sites, with the exception of M1 (Fig. 5E). In contrast, the previously reported SCas12a assay [9] only demonstrated significant performance at M7, M12, M16, and M18. Additionally, we have conducted experiments to monitor the specificity of the DTR assay for dsDNA targets. The results show that the DTR system (Supplementary Fig. S6) exhibits a specificity similar to that of the conventional Cas12a system, with only a slightly higher specificity observed at the M10–M14 sites.

Together, the DTR method has been demonstrated to be a highly sensitive and specific nucleic acid detection approach, with broad prospects for clinical applications.

### Cas12a DTR assay for clinical diagnostic applications

**Figure 6.**
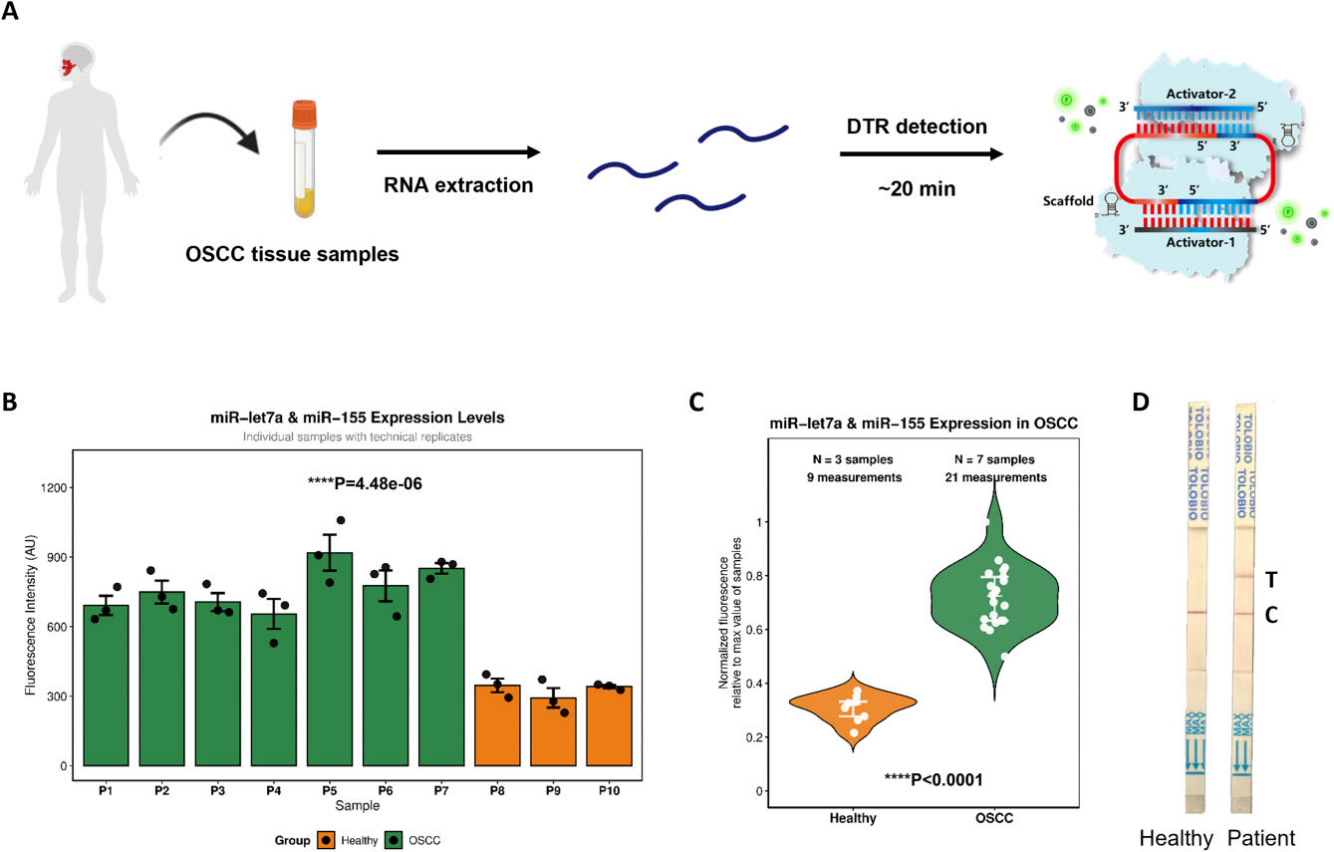
Cas12a DTR assay for clinical diagnostics. (**A**) Schematic outlining of RNA extraction from human OSCC tissue samples to dual detection of miR-let-7a and miR-155 by Cas12a DTR fluorescence assay. (**B**) Expression levels of miR-let7a and miR-155 in OSCC and healthy controls. Bar charts show mean expression levels of each sample, error bars indicate standard deviation, and scatter points represent technical replicates. OSCC group showed significantly higher expression than healthy controls (*P* < .001). AU: Arbitrary units. *n* = 10 samples (7 OSCC, 3 healthy controls). (**C**) Distribution of miR-let7a and miR-155 expressions in OSCC patients and healthy controls. Violin plots illustrate the complete data distribution shape, with white median lines indicating central tendency, white quartile lines showing data spread, and white dots representing actual measurements of each technical replicate. The OSCC group exhibited significantly higher expression levels compared to healthy controls (*P* < .0001). *n* = 30 measurements from 10 samples (7 OSCC, 3 healthy controls). (**D**) Detection of healthy and OSCC patient samples by colorimetric lateral flow assay to generate visible signals.

Cas12a DTR system provides the possibility to detect two RNAs simultaneously in the clinic to increase the accuracy of the single readout. OSCC is one of the most common head and neck malignancies, and the adequacy of surgical margins plays a critical role in determining the prognosis of the cancer [27–29]. However, currently, there remains an urgent need for effective intraoperative rapid detection methods for assessing resected tumor tissues. Previous studies have demonstrated the high-level expression of miR-let-7a and miR-155 in OSCC patient tumor tissues [28], ranging from picomolar to nanomolar concentrations, which falls within the dynamic range of the Cas12a DTR system. In our study, RNA was first extracted and purified from OSCC resection specimens and subsequently detected using the DTR fluorescence method within 20 min (Fig. 6A). The results (Fig. 6B and C) show that the DTR method effectively distinguishes patients with OSCC from healthy individuals. Moreover, by applying commercially available Cas12a LFA strips to clinical samples, we successfully identified positive cases among healthy controls (Fig. 6D). Overall, these findings highlight the excellent sensitivity and specificity of the Cas12a DTR method for disease diagnostics.

## Discussion

In this work, we introduced a novel topology-guided Cas12a DTR system, designed for noncontiguous target RNA/DNA activation. Unlike previous Cas12a-based tools focused on single-target analysis, the DTR system uses two split crRNA fragments and two Cas12a proteins that cooperatively reconstitute upon recognizing two nucleic acid activators. This innovative design allows specific dual detection of various nucleic acid targets in a single readout, overcoming limitations of traditional methods that required complex logic gates, microfluidic technology, or dynamic molecular circuits. The DTR system’s ability to function as a restricted AND-gate logic for dual RNA and DNA target detection provides new possibilities for more efficient and precise diagnostic applications.

Firstly, the proof-of-concept study showed that the DTR system effectively activates Cas12a’s *trans*-cleavage activity only when both target miRNAs are present, indicating its potential for simultaneous multi-miRNA target detection. Further optimization of break sites in the spacer region enhanced system performance, with the T8 + T12′ combination achieving the highest activation efficiency. This emphasizes the importance of precise topological assembly for optimal DTR system functionality. Additionally, we found that the DTR system exhibits a synergistic activation mode. Specifically, the Cas12a enzymatic activity in the DTR system is modulated by the combined concentrations of both activators rather than being strictly dictated by the lowest concentration of a single activator. This cooperative regulatory mechanism is crucial for efficient system activation and provides a foundation for downstream logic operations and experimental designs.

Secondly, the DTR system demonstrates high sensitivity for both RNA and DNA substrates. Moreover, this assay demonstrates high specificity for SNVs across all RNA target detection sites. This specificity, attributed to the DTR system’s stringent component assembly requirements, ensures only perfectly matched targets trigger Cas12a activation. This feature is vital for clinical applications where distinguishing between closely related nucleic acid sequences is essential for accurate diagnosis.

Finally, the disease diagnostic potential of the Cas12a DTR assay was demonstrated by detecting two OSCC-related miRNAs (miR-155 and miR-let-7a) in clinical samples. The assay’s ability to differentiate between healthy individuals and OSCC patients within 20 min highlights its potential as a rapid, accurate diagnostic tool. Successful use of commercial LFA for clinical sample testing further strengthens the assay’s practicality and feasibility for point-of-care applications. Despite its advantages, the DTR system has room for improvement. Its performance in detecting targets with varying concentrations and ratios requires further investigation to ensure reliability in real-world clinical scenarios. Additionally, exploring the DTR system’s applicability to a wider range of nucleic acid targets and its integration with other diagnostic platforms could enhance its utility and impact in molecular diagnostics.

In conclusion, the Cas12a DTR system is a powerful, innovative tool for nucleic acid detection, offering simultaneous, sensitive, and specific detection of both RNA and DNA targets. Its clinical potential, particularly in rapidly and accurately diagnosing diseases like OSCC, is promising. In future, we hope the DTR system will play a pivotal role in advancing nucleic acid detection technologies and improving clinical diagnostic outcomes.

## Supplementary Material

gkaf893_Supplemental_File

## Data Availability

The data underlying this article are available in the article and in its online supplementary material.
