# Supplementary material for: CRISPR/Cas12a DTR system: a topology-guided Cas12a assay for specific dual detection of RNA and DNA targets
Source: Nucleic Acids Res. 2025 Sep 10;53(17):gkaf893. doi: 10.1093/nar/gkaf893 (PMC12421382; doi:10.1093/nar/gkaf893)
Supplement: gkaf893_Supplemental_File [file gkaf893_supplemental_file.pdf]

CRISPR/Cas12a DTR System: A Topology-guided Cas12a Assay for Specific Dual Detection of RNA and DNA Targets

Qingyuan Jiang[1, †], Shuqi Jin[1, †], Zhichao Qin[2,+], Junqi Zhang[1], Ruyi He[2], Zhuo Chen[3], Bin Qiao[3], Jie Qiao[2]* and Yi Liu[1, 4]*

[1] State Key Laboratory of Biocatalysis and Enzyme Engineering, School of Life Sciences, Hubei University, Hubei 430042 (China)

[2] Pilot Base of Food Microbial Resources Utilization of Hubei Province, School of Life Science and Technology, Wuhan Polytechnic University, Hubei 430023 (China)

[3] Department of Oral and Maxillofacial Surgery, The First Affiliated Hospital of Zhengzhou University, Zhengzhou University, Zhengzhou 450001, China

[4] BravoVax Co., Ltd., Wuhan, Hubei 430075 (China)

*To whom correspondence should be addressed. Email: jieqiao@whpu.edu.cn or yiliu0825@hubu.edu.cn

[†] These authors contributed equally to the work.

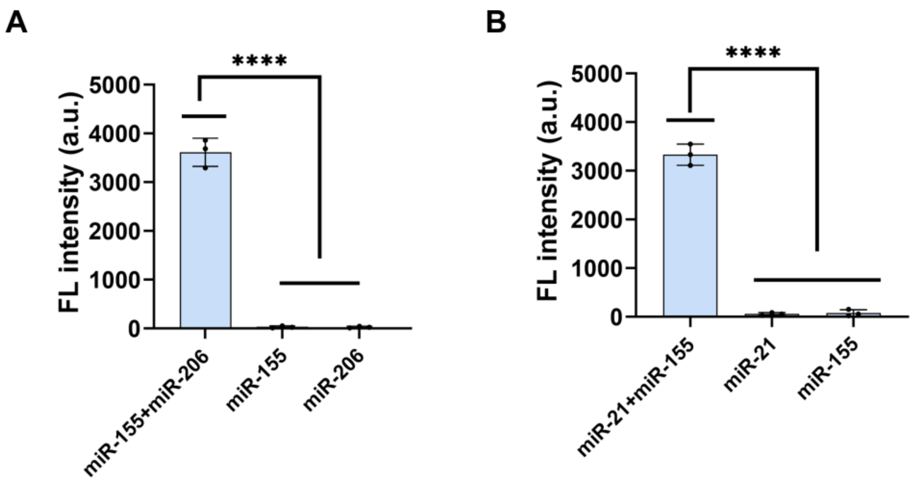

Figure S1. Specific dual detection of miRNAs by Cas12a DTR assay. (A) Detection of the combination of miR-155 and miR-206. **(B)** Detection of the combination of miR-21 and miR-155. Note: We have also tested other miRNAs, including miR-31, miR-222, and their combinations, and identical results were obtained.

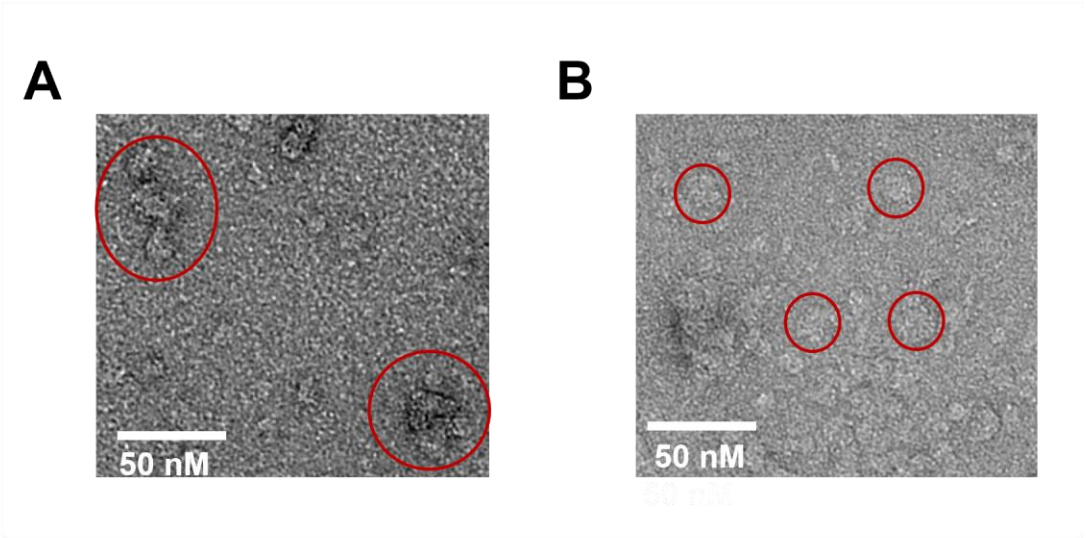

Figure S2. 120 kV cryo-EM images of the (A) Cas12a DTR system and **(B)** Control 2-1 system, as illustrated in Figure 2E.

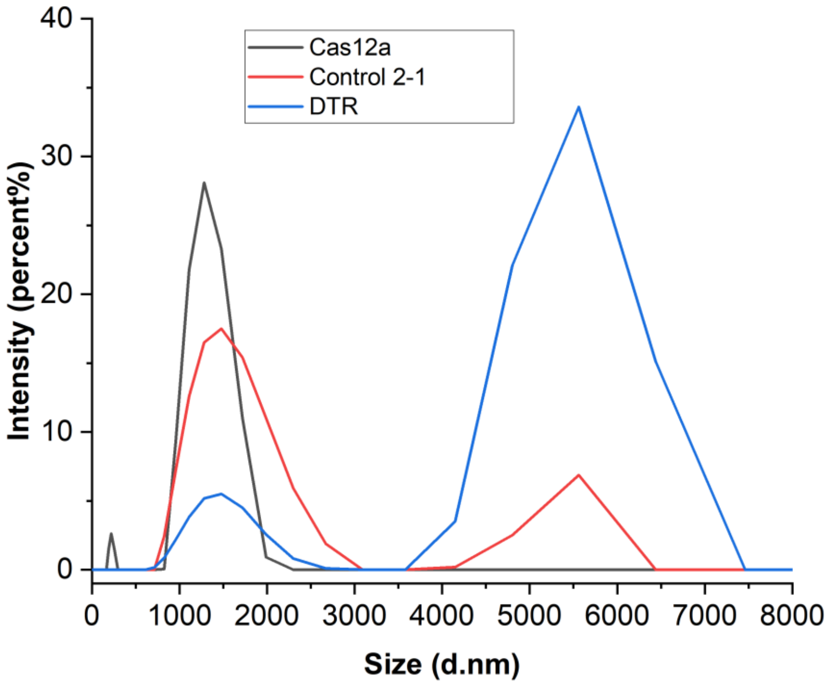

Figure S3. Dynamic light scattering (DLS) analysis experiment. The test was performed on the complexes, including the Cas12a protein, the complex from the Control 2-1 system, and the complex from the DTR system, as shown in Figure 2E.

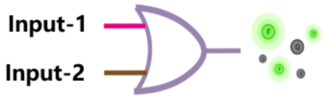

Input-1	Input-2	Output
0	0	0
1	0	1
0	1	1
1	1	1

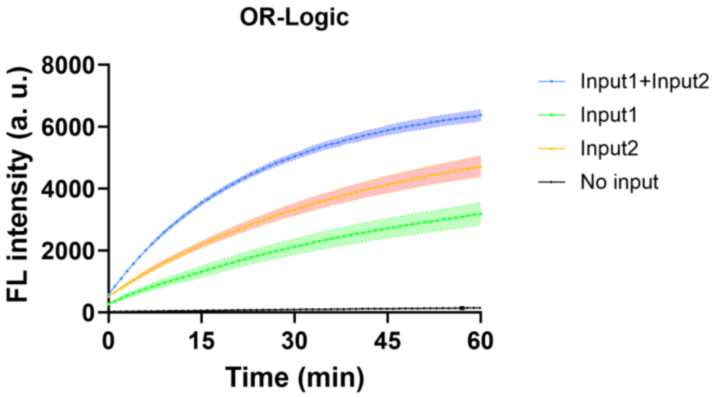

Figure S4. OR-logic detection of nucleic acids by Cas12a DTR system. Fluorescence analysis for the OR-logic detection of two miRNA inputs by DTR assay.

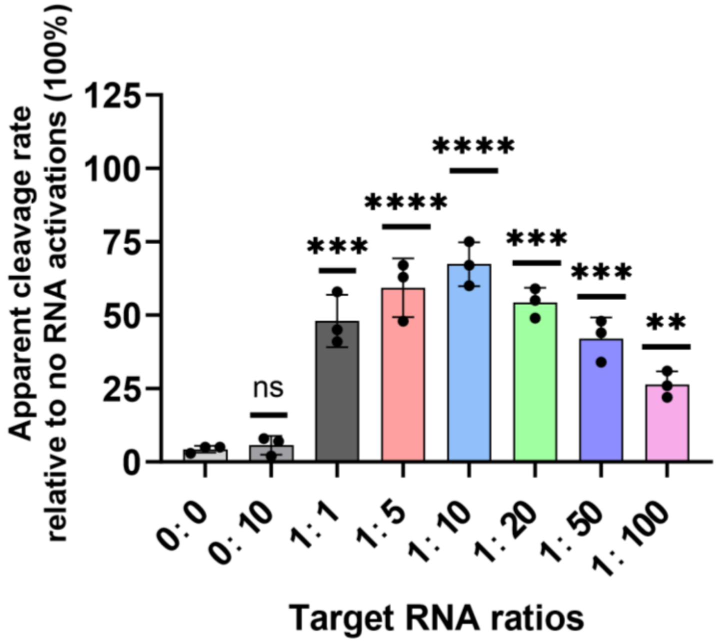

Figure S5. The Cas12a DTR system enables the discrimination of samples containing two miRNA targets at varying concentration ratios. The concentration of miRNA-1 was maintained at 1 pM while the concentration of miRNA-2 was varied, as determined by a two-tailed t-test. Data are presented as mean values ± standard deviation from three independent experiments.

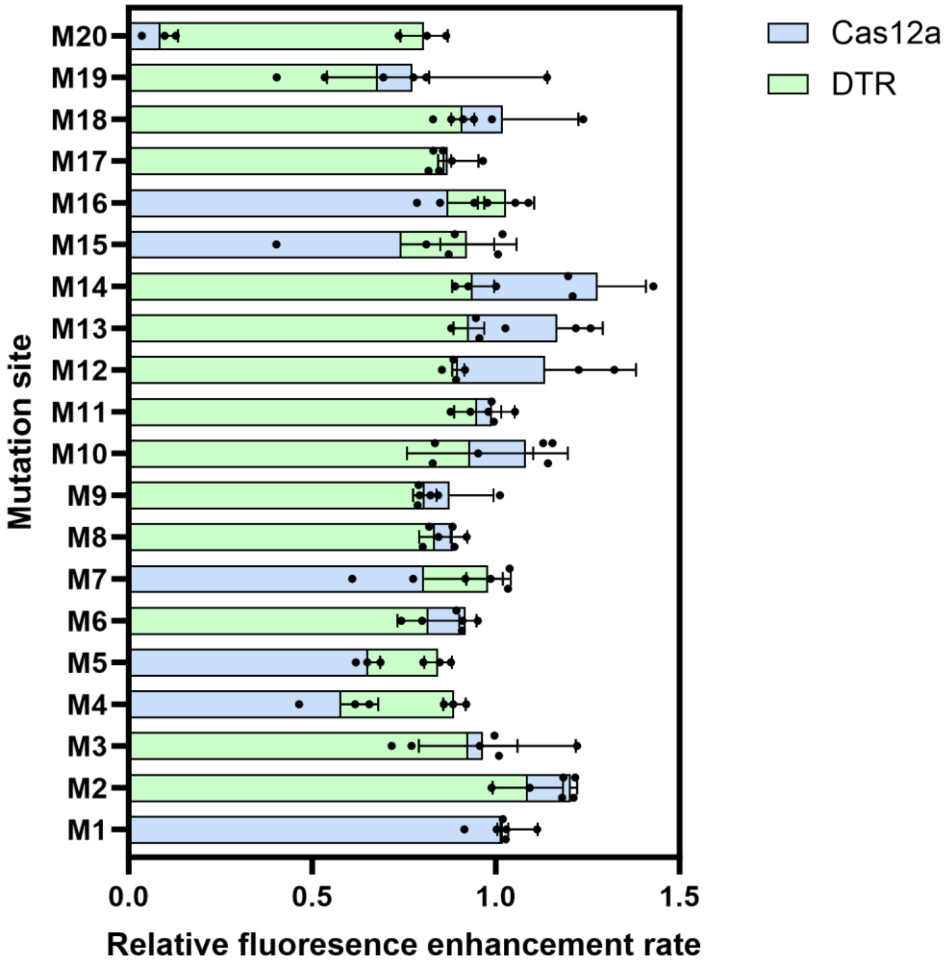

Figure S6. Determination of specificity of the DTR assay for dsDNA. Comparison of fluorescence fold changes in the *trans*-cleavage assay between Cas12a and DTR utilizing dsDNA substrate. All fluorescence values were normalized to those of WT activator. Statistical analysis for n=3 biologically independent replicates comparing the normalized fold change for SCas12aV vs DTR assay. Statistical analysis was conducted using a two-tailed t-test. Statistical significance was determined as follows: ns (not significant) for $p > 0.05$, * for $p \leq 0.05$, ** for $p \leq 0.01$, *** for $p \leq 0.001$, and **** for $p \leq 0.0001$. error bars represent mean value +/− SD (n =3).

Table S1: List of Spacer and DNA activators used in this study (5'→3').

Name	Sequence
Spacer: S0	AAUUUCUACUAAGUGUAGAU

miR-155+miR-let-7a: ssDNA acivator

6+14 -A1	AACCTACTACCTCAAACCCC
6+14 -A2	ACGATTAGCATTAAAACTAT
7+13-A1	ACCTACTACCTCAAACCCCT
7+13-A2	CGATTAGCATTAAAACTATA
8+12-A1	CCTACTACCTCAAACCCCTA
8+12-A2	GATTAGCATTAAAACTATAC
9+11-A1	CTACTACCTCAAACCCCTAT
9+11-A2	ATTAGCATTAAAACTATACA
10+10-A1	TACTACCTCAAACCCCTATC
10+10-A2	TTAGCATTAAAACTATACAA
11+9-A1	ACTACCTCAAACCCCTATCA
11+9-A2	TAGCATTAAAACTATACAAC
12+8-A1	CTACCTCAAACCCCTATCAC
12+8-A2	AGCATTAAAACTATACAACC
13+7-A1	TACCTCAAACCCCTATCACG
13+7-A2	GCATTAAAACTATACAACCT
14+6-A1	ACCTCAAACCCCTATCACGA
14+6-A2	CATTAAAACTATACAACCTA

miR-155+miR-let-7a: hybrid DNA acivator

S6.1-PAM	GGTGGAATTCTGCAGTTTG
ds-8+12-155-7a-A1- F	CCTACTACCTCAAACCCCTACAAACTGCAGAATTCCACC
ds-8+12-155-7a-A1-R	GGTGGAATTCTGCAGTTTGTAGGGGTTTGAGGTAGTAGG
ds-8+12-155-7a-A2-F	GATTAGCATTAAAACTATACCAAACTGCAGAATTCCACC
ds-8+12-155-7a-A2-R	CTAATCGTAATTTTGATATGGTTTGACGTCTTAAGGTGG
S6.1-8+12-155+7a-R-A1	CCTACTACCTCAAACCCCTACAAACTGCAGAATTCCACC
S6.1-8+12-155+7a-R-A2	GATTAGCATTAAAACTATACCAAACTGCAGAATTCCACC

ds-PIK3CA	GTCCCATAGTCATGCATTGTTTTGCACCCCAAATTTTTTATTGTTCATAGCAGCATGGT
ds-PIK3CA-R	ACCATGCTGCTATGAACAATAAAAAATTTGGGGTGCAAAACAATGCATGACTATGGGAC

Determination of SNVs by DTR assay

8+12-155-7a-A1-WT	CCTACTACCTCAAACCCCTA
8+12-155-7a-A1-M1	GCTACTACCTCAAACCCCTA
8+12-155-7a-A1-M2	CGTACTACCTCAAACCCCTA
8+12-155-7a-A1-M3	CCAACTACCTCAAACCCCTA
8+12-155-7a-A1-M4	CCTTCTACCTCAAACCCCTA
8+12-155-7a-A1-M5	CCTAGTACCTCAAACCCCTA
8+12-155-7a-A1-M6	CCTACAACCTCAAACCCCTA
8+12-155-7a-A1-M7	CCTACTTCCTCAAACCCCTA
8+12-155-7a-A1-M8	CCTACTAGCTCAAACCCCTA
8+12-155-7a-A1-M9	CCTACTACGTCAAACCCCTA
8+12-155-7a-A1-M10	CCTACTACCACAAACCCCTA
8+12-155-7a-A1-M11	CCTACTACCTGAAACCCCTA
8+12-155-7a-A1-M12	CCTACTACCTCTAACCCCTA
8+12-155-7a-A1-M13	CCTACTACCTCATACCCCTA
8+12-155-7a-A1-M14	CCTACTACCTCAATCCCCTA
8+12-155-7a-A1-M15	CCTACTACCTCAAAGCCCTA
8+12-155-7a-A1-M16	CCTACTACCTCAAACGCCTA
8+12-155-7a-A1-M17	CCTACTACCTCAAACCGCTA
8+12-155-7a-A1-M18	CCTACTACCTCAAACCCGTA
8+12-155-7a-A1-M19	CCTACTACCTCAAACCCCAA
8+12-155-7a-A1-M20	CCTACTACCTCAAACCCCTT

Investigation of the university of DTR assay

8+12-155-206-A1	CCTTACATTCCAAACCCCTA
8+12-155-206-A2	GATTAGCATTAACCACACAC
8+12-155-21-A1	TCTGATAAGCTAAACCCCTA
8+12-155-21-A2	GATTAGCATTAATCAACATC
8+12-206-155-A1	GATTAGCATTAACCACACAC
8+12-206-155-A2	CCTTACATTCCAAACCCCTA
8+12-7a-21-A1	TCTGATAAGCTAAACTATAC
8+12-7a-21-A2	CCTACTACCTCATCAACATC
8+12-7a-206-A1	CCTTACATTCCAAACTATAC
8+12-7a-206-A2	CCTACTACCTCACCACACAC
8+12-21-206-A1	CCTTACATTCCATCAACATC
8+12-21-206-A2	CCTTACATTCCATCAACATC

8+12-7a-222-A1	CCAGATGTAGCTAACTATAC
8+12-7a-222-A2	CCTACTACCTCAACCCAGTA
8+12-155-31-A1	AGCATCTTGCCTAACCCCTA

Table S2 List of Target RNA used in this study (5'→3')

miR -155	UUAAUGCUAAUCGUGAUAGGGGUU
miR -let-7a	UGAGGUAGUAGGUUGUAUAGUU
miR -206	UGGAAUGUAAGGAAGUGUGUGG
miR -21	UAGCUUAUCAGACUGAUGUUGA
miR-222	AGCUACAUCUGGCUACUGGGU
miR-31	AGGCAAGAUGCUGGCAUAGCU
